# Infantile Mortality

**Published:** 1906-08-25

**Authors:** 


### Infantile Mortality.

During the next few weeks the mortality amongst infants will probably be very high. Towards the end of every summer the disease that has been called Cholera infantum carries off its thousands. Alarmingly common though this disease is it can hardly be said that its precise causation is definitely known. A bacillus similar to that which causes tropical dysentery is considered by many investigators to be mainly responsible, but this point can scarcely be said to be definitely proved. Undoubtedly, however, some micro-organism must be the main offending agent, but, accepting this as indisputable, its method of entry into the infant's body has to be considered. Probably the micro-organism obtains entrance in more than one way, but it seems to be clear that the child must become infected in the great majority of instances through the medium of its food. Although infants at the breast occasionally suffer from and succumb to acute diarrhœa, the disease claims its victims almost entirely from among those that are artificially fed. If breast-fed babies escape while bottle-fed babies die, either the breast-fed babies must present greater resistance to the disease or the bottle-fed babies must be exposed to greater danger of receiving it. Possibly the safety of the infant at the breast is to be explained in some measure on both of these grounds. The infant nursed by the mother is not only less exposed to but more able to withstand attack. Professor Byers, in an interesting address before the Bangor Nursing Society, recently referred to many details in connection with infant mortality, and laid stress upon the importance of nursing by the mother. He mentioned, among other valuable facts which have come to us from our French neighbours,, that the great woollen and cloth manufacturers, MM. Blin and Blin, of Elbeuf, near Rouen, decided to deposit 100 francs in the savings bank for each of their employés who nourished their children themselves for twelve months after birth. Formerly no woman in their employ nursed her baby. As a result of this arrangement from April 15, 1904, to September 1, 1905, 37.5 per cent. of the infants were breast-fed and 62.5 per cent. were bottle-fed. During the same period the mortality amongst the breast-fed babies was nil, while amongst those artificially fed it was 21 per 100. Professor Byers also quoted facts relating to the village of Villiers le duc, which we have previously mentioned, but are worth repeating. In that village for a century the death-rate of infants under one year had never fallen below 130 per 1,000 and had risen to 288 per 1,000, but after methods had been adopted, which encouraged mothers to nurse their own infants, no death occurred under the age of one year during a period of ten years.

Coming to our own country statistics have been collected which thoroughly support the experience of those who have been endeavouring to diminish infant mortality in France. In Birmingham the medical officer of health, dealing with the statistics of 3,000 babies, found that among the breast-fed the mortality was 8 per 1,000, among infants artificially fed 25.2 per 1,000. In Huddersfield, also, it has been found that for one baby that dies among those nursed at the breast fifteen die among those artificially fed. Facts such as these need little comment. It is clear that departure from Nature's methods of providing for man in his stage of most complete helplessness entails grave danger to his race. And it is at this time of year that the greatest mortality occurs. As has been mentioned, the causation of summer diarrhœa is not yet perfectly clear, but it is a fact beyond dispute that the disease mainly attacks bottle-fed infants. Where artificial feeding is necessary all water and milk given to infants should, therefore, especially at this season of the year, be boiled. This may not be an infallible preventive of infection from this dire disease, but it greatly diminishes the risk such feeding entails.

